# Drains result in greater reduction of subdural width and midline shift in burr hole evacuation of chronic subdural haematoma

**DOI:** 10.1007/s00701-020-04356-z

**Published:** 2020-04-27

**Authors:** Laurence Johann Glancz, Michael Tin Chung Poon, Peter John Hutchinson, Angelos Georgiou Kolias, Paul Martin Brennan, on behalf of the British Neurosurgical Trainee Research Collaborative (BNTRC)

**Affiliations:** 1 Department of Neurosurgery, Queen’s Medical Centre, Nottingham, UK; 2 Translational Neurosurgery, Centre for Clinical Brain Sciences, University of Edinburgh, Edinburgh, UK; 3 Usher Institute, University of Edinburgh, Edinburgh, UK; 4 Division of Neurosurgery, Department of Clinical Neurosciences, University of Cambridge and Addenbrooke’s Hospital, Cambridge, UK; 5 Surgery Theme, Cambridge Clinical Trials Unit, Cambridge Biomedical Campus, Cambridge, UK

**Keywords:** Burr hole evacuation, Chronic subdural haematoma, Drains, Midline shift, Outcomes, Radiology

## Abstract

**Background:**

Drain insertion following chronic subdural haematoma (CSDH) evacuation reduces recurrence and improves outcomes. The mechanism of this improvement is uncertain. We assessed whether drains result in improved postoperative imaging, and which radiological factors are associated with recurrence and functional outcome.

**Methods:**

A multi-centre, prospective cohort study of CSDH patients was performed between May 2013 and January 2014. Patients aged > 16 years undergoing burr hole evacuation of primary CSDH with pre- and postoperative imaging were included in this subgroup analysis. Baseline and clinical details were collected. Pre- and postoperative maximal subdural width and midline shift (MLS) along with clot density were recorded. Primary outcomes comprised mRS at discharge and symptomatic recurrence requiring re-drainage. Comparisons were made using multiple logistic regression.

**Results:**

Three hundred nineteen patients were identified for inclusion. Two hundred seventy-two of 319 (85%) patients underwent drain insertion at the time of surgery versus 45/319 (14%) who did not. Twenty-nine of 272 patients who underwent drain insertion experienced recurrence (10.9%) versus 9 of 45 patients without drain insertion (20.5%; *p* = 0.07). Overall change in median subdural width was significantly greater in the drain versus ‘no drain’ groups (11 mm versus 6 mm, *p* < 0.01). Overall change in median midline shift (MLS) was also significantly greater in the drain group (4 mm versus 3 mm, *p* < 0.01). On multivariate analysis, change in maximal width and MLS were significant predictors of recurrence, although only the former remained a significant predictor for functional outcome.

**Conclusions:**

The use of subdural drains results in significantly improved postoperative imaging in burr hole evacuation of CSDH, thus providing radiological corroboration for their recommended use.

**Electronic supplementary material:**

The online version of this article (10.1007/s00701-020-04356-z) contains supplementary material, which is available to authorized users.

## Introduction

Chronic subdural haematoma (CSDH) is increasingly common in neurosurgical practice, especially with our ageing population; the incidence has been estimated to be 8.2 to 14.0 per 100,000 person-years [17]. Surgery is the mainstay for management of symptomatic haematomas, but the exact operative strategy and perioperative management varies greatly [3]. Level 1 evidence suggests the placement of a closed subdural drainage system at the time of burr hole evacuation maintained for 48 h postoperatively reduces symptomatic recurrence [23]; it also improves long-term outcomes [13]. We have reported in a large prospective national UK audit that drains are indeed associated with a significantly lower recurrence rate and better early functional outcome [3]. We have also reported separately that recurrence rates were comparable between subdural (7.7%) and subgaleal (9.1%) groups (*p* = 0.95) [11]. Nevertheless, this is a contentious area, with a recent randomised trial reporting a lower, although not statistically significant, recurrence rate with subgaleal drains [27] and another recent national cohort study from Denmark reporting that subdural drains were associated with lower recurrence [1].

There are few reports in the literature as to whether drains result in better postoperative imaging and how they mediate improved outcomes. Radiological predictors of recurrence have been examined extensively but with conflicting results. There is some evidence to suggest that smaller postoperative residual subdural collections with less midline shift are associated with lower recurrence. There are very few studies that have evaluated radiological predictors of functional outcome. We therefore sought to evaluate radiological findings in patients with and without postoperative drains in a post hoc subgroup analysis of our large prospective cohort study. Our secondary aim was to examine the relationship of radiological parameters with (i) recurrence and (ii) early functional outcome.

## Methods

### Participants and study settings

**Fig. 1:**
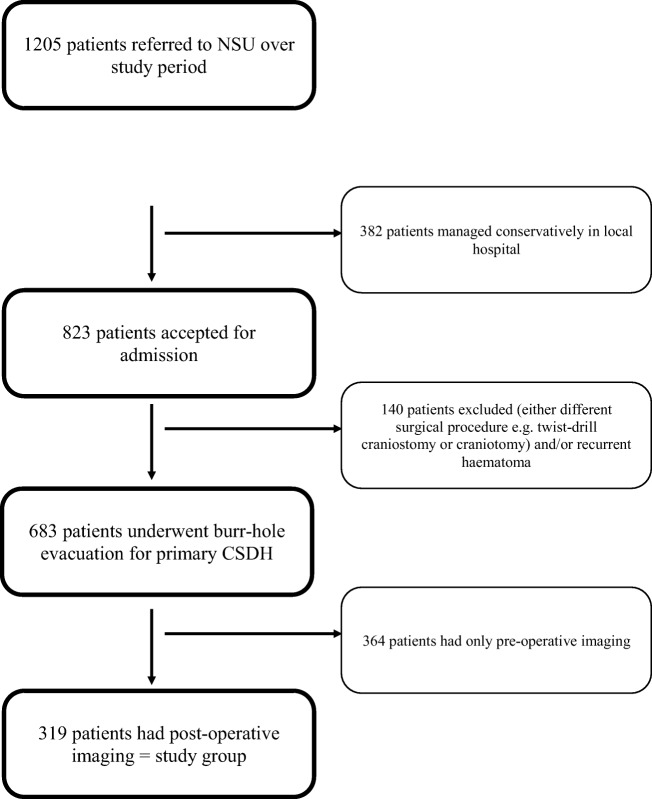
Flow chart showing patient selection in this study

Our study methodology has already been extensively described [3, 7]. In summary, we conducted a multi-centre, prospective cohort study assessing the variation in operative and perioperative strategies for CSDH along with clinical outcomes. Study participants were identified and enrolled at 26 of the 33 UK and Ireland neurosurgical units (NSUs) between May 1, 2013 and January 31, 2014. Data collection periods within these dates varied between NSUs with an average timeframe of 153 days (range 76–241 days). There was no obvious giant contributing NSU (see supplementary table 1). Eligibility criteria were age > 16 years, presentation with a primary or recurrent CSDH confirmed on cranial imaging, and referral to a participating NSU. Data were collected for 1205 patients with CSDH referred to the 26 participating NSUs; recruitment per unit ranged from 4 to 175 patients (mean 46 patients). Of 1205 patients referred, 823 (68.3%) were accepted for NSU admission. In the remaining 382 patients, CSDH was managed at their referring hospital. The most common reason for not being transferred was that the subdural collection was considered small and insufficient to explain a patient’s symptoms, or that the patient was asymptomatic. Of these patients, only those who were transferred to an NSU and underwent burr hole evacuation of index CSDH with pre- and postoperative imaging were included in this subgroup analysis. Therefore, all patients who had undergone drainage via an alternative surgical technique (e.g. craniotomy or twist-drill craniotomy) and/or previous drainage of an ipsilateral CSDH were excluded. After these exclusion criteria were applied, we had a total population of 319 patients for this subgroup analysis (see Fig. 1). A further analysis was performed including those patients undergoing burr hole evacuation of index CSDH including patients who lacked postoperative imaging (see supplementary tables 2–8c); these patients were otherwise subject to the same aforementioned exclusion criteria.

### Data collection and outcome measures

Data were collected through the British Neurosurgical Trainee Research Collaborative, which is a network of neurosurgical trainees and supervising consultants in each UK and Ireland NSU [4]. Local trainee investigators identified patients at the time of admission to the NSU from on-call referral databases or operating theatre logbooks. Patient demographic data, baseline characteristics including medical comorbidities and relevant medication history, and details of pre-, intra-, and postoperative management were recorded by local clinical teams. For all patients, haematoma laterality, maximum subdural width, maximum midline shift (MLS), clot density (hypodense, isodense or mixed), and presence or absence of membranes were assessed on preoperative imaging and recorded. The majority of neurosurgeons in the UK do not request routine postoperative imaging [24]. If postoperative imaging was obtained, we recorded the date, indication for imaging (routine or due to clinical concern), maximum subdural width and maximum midline shift. For patients with both pre- and postoperative imaging, we were able to calculate change in maximum subdural width and change in maximum midline shift.

Reoperation within 60 days of index admission was identified and recorded; this formed our definition of recurrence. The decision to proceed with revision surgery was at the discretion of the patient’s consultant neurosurgeon, based on clinical symptoms, correlated with imaging. The mRS score on day of discharge and discharge destination from the NSU, as well as morbidity and mortality and length of stay in the NSU were also recorded by local clinical teams. The following mRS was used: 0, no symptoms; 1, no significant disability (able to carry out all usual activities, despite some symptoms); 2, slight disability (able to look after own affairs without assistance, but unable to carry out all previous activities); 3, moderate disability (requires some help, but able to walk unassisted); 4, moderately severe disability (unable to attend to own bodily needs without assistance, and unable to walk unassisted); 5, severe disability (requires constant nursing care and attention, bedridden, incontinent); 6, dead.

Data were submitted to a secure online database maintained by the Outcome Registry Intervention and Operation Network (ORION) at the University of Cambridge. Each NSU was the data controller for its own data. Local governance approvals were in place in each participating NSU. Individual patient consent was not required and therefore not sought for this study. The study protocol was approved and supported by the Academic Committee of the Society of British Neurological Surgeons (SBNS).

### Statistical analysis

We used parametric and non-parametric tests to compare baseline demographic, clinical and radiological characteristics between patients with and without drain insertion. Our first description of this cohort showed that drain insertion and preoperative GCS to be associated with recurrence [3]. For investigating the influence of radiological factors on the risk of recurrence, we formed three multivariate analyses using multiple logistic regression: (i) variables included drain insertion, preoperative GCS; (ii) addition of change in maximal width; (iii) further addition of change in maximal shift. We used change in maximal width and maximal shift as variables because the aim of operation is to achieve these and they are more meaningful measures than absolute values. Functional outcome according to mRS was dichotomised into favourable (mRS 0–3) and unfavourable (mRS 4–6) outcomes. We adopted a similar approach for regression on unfavourable functional outcome. The variables included in the first model were age, preoperative mRS, preoperative GCS, drain inserted and postoperative bed rest. Patients with missing data were excluded if the missing data were relevant to the particular analysis. A *p* value of < 0.05 denoted statistical significance. We used Stata version 13.0 (StataCorp) for all analyses.

## Results

### Drain versus no drain

Baseline and perioperative characteristics of 319 patients with pre- and postoperative imaging are illustrated in Tables 1 and 2, respectively. Patients who underwent drain insertion were found to be significantly older and have poorer preoperative mRS scores compared with those who did not undergo drain insertion. Significant differences were also observed in bed rest prescribed, and number of burr holes used between groups; the ‘no drain’ group underwent single burr hole evacuation more frequently in comparison to the drain group (22.2% versus 4.4%, *p* < 0.01).

**Table 1:** Baseline characteristics of 319 patients who underwent burr hole evacuation of CSDH and had postoperative imaging

	Total (*n* = 319)	No drain inserted* (*n* = 45)	Drain* (*n* = 272)	*p* value**
Age (median; IQR)	77 (66–84)	71 (56–81)	78 (67–85)	< 0.01
Gender				0.62
Female	102 (32.0)	14 (31.1)	88 (32.4)	
Male	217 (68.0)	31 (68.9)	184 (67.7)	
Medical comorbidities				
Diabetes mellitus	54 (16.9)	7 (15.6)	47 (17.3)	0.78
Dementia	29 (9.1)	3 (6.7)	26 (9.6)	0.53
COPD	17 (5.3)	1 (2.2)	16 (5.9)	0.31
Cerebrovascular event	58 (18.2)	8 (17.8)	50 (18.4)	0.92
Ischaemic heart disease	77 (24.1)	9 (20.0)	67 (24.6)	0.50
Arrhythmia	66 (20.7)	11 (24.4)	55 (20.2)	0.52
Epilepsy	20 (6.3)	4 (8.9)	15 (5.5)	0.38
CSF shunt	7 (2.2)	1 (2.2)	6 (2.2)	0.99
Malignancy	25 (7.8)	3 (6.7)	21 (7.7)	0.80
Metallic heart valve	9 (2.8)	2 (4.4)	7 (2.6)	0.48
History of head injury in past 3 months	192 (60.2)	26 (27.8)	164 (60.3)	0.75
Prior antithrombotic use	138 (43.3)	18 (40.0)	119 (43.8)	0.64
Prior antiplatelet use	75 (23.5)	13 (28.9)	61 (22.4)	0.34
Prior warfarin use	64 (20.1)	5 (11.1)	59 (21.7)	0.10
Other antithrombotics	4 (1.3)	1 (2.2)	3 (1.1)	0.53

*Two patients had missing data on drain insertion

**p value of chi-squared test or non-parametric test, when appropriate, comparing between patients with and without drain insertion

**Table 2:** Perioperative clinical characteristics of 319 patients who underwent burr hole evacuation of CSDH and had postoperative imaging

	Total (*n* = 319)	No drain inserted* (*n* = 45)	Drain* (*n* = 272)	*p* value
Preoperative platelet transfusion	27 (8.5)	2 (4.4)	25 (9.2)	0.29
Preoperative vitamin K	58 (18.2)	5 (11.1)	52 (19.1)	0.20
Preoperative FFP	8 (2.5)	0 (0)	7 (2.6)	0.28
Preoperative GCS (median, IQR)		14 (13–15)	14 (13.5–15)	0.63
GCS 3–8	17 (5.3)	2 (4.4)	15 (5.5)	
GCS 9–12	41 (12.9)	6 (13.3)	35 (12.9)	
GCS 13–15	261 (81.8)	37 (82.2)	222 (81.6)	
Preoperative mRS				0.04
mRS 0–3	183 (57.4)	32 (71.1)	149 (54.8)	
mRS 4–5	136 (42.6)	13 (28.9)	123 (45.2)	
CSDH laterality				0.11
Left	121 (37.9)	24 (53.3)	97 (36.7)	
Right	102 (32.0)	11 (24.4)	91 (34.5)	
Bilateral	86 (27.0)	10 (22.2)	76 (28.8)	
Unknown/missing	10 (3.1)	-	-	
Timing of operation				0.20
Within 2 days of referral	223 (69.9)	16 (36.4)	72 (27.0)	
After 2 days of referral	88 (27.6)	28 (63.6)	195 (73.0)	
Unknown/missing	8 (2.5)	-	-	
Number of burr hole(s)				< 0.01
Single burr hole	22 (6.9)	10 (22.2)	12 (4.4)	
> 1 burr holes	295 (92.5)	35 (77.8)	260 (95.6)	
Unknown/missing	2 (0.6)	-	-	
Postoperative bed rest				0.05
No specific instructions	119 (37.3)	24 (53.3)	95 (34.9)	
1–12 h	27 (8.5)	6 (13.3)	21 (7.7)	
12–24 h	118 (37.0)	11 (24.4)	107 (39.3)	
24–48 h	46 (14.4)	4 (8.9)	42 (15.4)	
48+ hours	7 (2.2)	0 (0)	7 (2.6)	
Unknown/missing	2 (0.6)	-	-	
Postoperative imaging				0.10
Indication not specified	2 (0.6)			
Routine	184 (57.7)	21 (46.7)	163 (59.9)	
Due to concerns	133 (41.7)	24 (53.3)	109 (40.1)	

*Two patients had missing data on drain insertion

Overall recurrence rate observed was 11.9% (38 of 319 patients). The median time to recurrence was 12.5 days. Nine of 45 patients without drain insertion experienced recurrence (20.5%) compared with 29 of 272 patients who underwent drain insertion (10.9%; *p* = 0.07). The timing of postoperative CT scan was not significantly different between the two groups; the median for both groups was 2 days postsurgery (*p* = 0.5). One hundred ninety-three of 311 patients with data had a scan within 2 days of the operation.

Pre- and postoperative radiological characteristics for both groups are shown in Table 3. Patients with unilateral drained haematomas receiving drains had significantly greater haematoma width with greater MLS on preoperative imaging. Overall change in median subdural width was significantly greater in the drain versus ‘no drain’ groups (11 mm versus 6 mm, *p* < 0.01). This difference was observed in unilateral cases (*p* < 0.01) but did not reach significance when considering bilateral haematomas separately (*p* = 0.08). Change in median MLS was also significantly greater in the drain group (*p* < 0.01).

**Table 3:** Comparison of pre- and postoperative radiological characteristics of 319 CSDH patients undergoing burr hole evacuation with and without drain insertion

	Total (*n* = 319)	No drain inserted* (*n* = 45)	Drain* (*n* = 272)	*p* value
CSDH density on initial CT scan				0.27
Hypodense	89 (27.9)	17 (37.8)	71 (26.1)	
Isodense	60 (18.8)	7 (15.6)	53 (19.5)	
Mixed	170 (53.3)	21 (46.7)	148 (54.4)	
Presence of membrane on CT scan				0.22
Yes	133 (41.7)	15 (33.3)	117 (43.0)	
No	186 (58.3)	30 (66.7)	155 (57.0)	
Preoperative maximal width (mm)				
Overall	25 (18–32)	20 (14–26)	25 (19–32)	< 0.01
Unilateral cases	22 (17–28)	20 (12–23)	23.5 (18–29)	< 0.01
Bilateral cases	34 (25–42)	33.5 (19–37)	34 (25–43.5)	0.27
Preoperative midline shift (mm)				
Overall	8 (4–11)	7 (4–10)	8 (4–12)	0.09
Unilateral cases	9 (6–12)	7 (5–11)	9.5 (6–13)	0.01
Bilateral cases	3 (0–6)	3 (0–7)	3 (0–6)	0.67
Time to operation (days)	1 (1–3)	2 (1–6)	1 (1–3)	0.09
Days between operation and postoperative scan (median; IQR)	2 (1–4)	2 (1–3.5)	2 (1–4)	0.50
Postoperative maximal width (mm)				
Overall	14 (9–21)	15 (10–22)	14 (9–21)	0.36
Unilateral cases	13 (8–19)	15 (9–22)	13 (8–18.5)	0.14
Bilateral cases	20 (12.29)	20 (12–28)	20.5 (12.5–29)	0.93
Change in maximal width (mm)				
Overall				
Median (IQR)	10 (5–15)	6 (2–9)	11 (6–16)	< .01
Reduced width	288 (90.9)	35 (77.8)	253 (93.0)	
Same or increased width	29 (9.2)	10 (22.2)	19 (7)	
Unilateral cases				
Median (IQR)	9 (5–14)	4 (0–8)	10 (6–15)	< 0.01
Reduced width	202 (90.6)	26 (74.3)	176 (93.6)	
Same or increased width	21 (9.4)	9 (25.7)	12 (6.4)	
Bilateral cases				
Median (IQR)	13 (7–20)	8 (6–9)	14 (7–20)	0.08
Reduced width	79 (91.9)	9 (90.0)	70 (92.1)	
Same or increased width	7 (8.1)	1 (10.0)	6 (7.9)	
Change in midline shift (mm)				
Overall				
Median (IQR)	4 (1–6)	3 (0–4)	4 (2–7)	< 0.01
Reduced shift	256 (80.8)	32 (71.1)	224 (82.4)	
Same or increased shift	61 (19.2)	13 (28.9)	48 (17.7)	
Unilateral cases				
Median (IQR)	0 (0–1)	3 (0–4)	5 (2–8)	< 0.01
Reduced shift	192 (86.1)	25 (71.4)	167 (88.8)	
Same or increased shift	31 (13.9)	10 (28.6)	21 (11.2)	
Bilateral cases				
Median (IQR)	0 (0–1)	2.5 (0–5)	2 (0–4)	0.84
Reduced shift	56 (65.1)	7 (70.0)	49 (64.5)	
Same or increased shift	30 (34.9)	3 (30.0)	27 (35.5)	

A supplementary analysis of pertinent features comparing patients with postoperative imaging and those with preoperative imaging alone (i.e. those who were excluded) revealed no significant difference in age, preop GCS, time to surgery, drain insertion, preoperative maximum width and MLS (see supplementary table 2). Recurrence rate was found to be higher in the group with postoperative imaging (12.2%) versus those with preoperative imaging alone (6.5%; *p* = 0.012).

### Radiological predictors of recurrence and functional outcome

On univariate analysis, postoperative maximal MLS/width and change in maximal subdural width and MLS were all found to be significantly associated with recurrence (Table 4). The same variables were found to be significantly associated with functional outcome (Table 4). On multivariate analysis, change in maximal width and MLS remained significant predictors of recurrence (Table 5), although only the former remained a significant predictor for functional outcome (Table 6).

**Table 4:** Unadjusted odds ratio for recurrence and unfavourable functional outcome at discharge in 319 patients with postoperative imaging

	Recurrence			Unfavourable functional outcome		
	OR	95% CI	*p* value	OR	95% CI	*p* value
Density on CT						
Hypodense	Ref	-	-	Ref	-	-
Isodense	1.34	0.45–3.94	0.60	0.57	0.25–1.32	0.18
Mixed	1.55	0.66–3.63	0.31	1.30	0.73–2.31	0.38
Presence of membrane on CT scan	1.28	0.65–2.54	0.47	1.10	0.67–1.82	0.71
Preoperative maximal width	1.01	0.98–1.04	0.68	0.99	0.97–1.01	0.48
Preoperative maximal shift	1.05	0.98–1.12	0.18	1.03	0.98–1.08	0.24
Postoperative maximal width	1.07	1.04–1.11	< 0.01	1.02	1.00–1.05	0.04
Postoperative maximal shift	1.28	1.18–1.40	< 0.01	1.13	1.06–1.20	< 0.01
Change in maximal width	0.92	0.88–0.95	< 0.01	0.95	0.92–0.98	< 0.01
Change in maximal shift	0.86	0.79–0.93	< 0.01	0.93	0.88–0.99	0.02

**Table 5:** Adjusted odds ratios for recurrence within 60 days using multiple logistic regression model based on 311 patients with postoperative imaging

	OR	95% CI	*p* value
Drain inserted	0.89	0.35–2.25	0.80
Preoperative GCS (13–15)	0.32	0.15–0.66	< 0.01
Change in maximal thickness (mm)	0.94	0.89–0.99	0.02
Change in maximal shift (mm)	0.90	0.82–0.99	0.03

**Table 6:** Adjusted odds ratios for unfavourable functional outcome at discharge using multiple logistic regression model based on 311 patients with postoperative imaging

	OR	95% CI	*p* value
Age	1.07	1.03–1.10	< 0.01
Preoperative mRS (mRS 4–5)	7.56	3.64–15.7	< 0.01
Preoperative GCS (13–15)	0.58	0.29–1.18	0.13
Drain inserted	0.50	0.19–1.34	0.17
> 1 burr hole	0.41	0.13–1.29	0.13
Postoperative bed rest			
1–12 h	1.43	0.40–5.11	0.59
12–24 h	1.53	0.74–3.18	0.25
24–48 h	1.59	0.63–3.98	0.32
> 48 h	4.11	0.48–35.4	0.20
Change in maximal thickness (mm)	0.93	0.89–0.98	< 0.01
Change in maximal shift (mm)	0.96	0.89–1.03	0.28

For multivariate analysis, we chose to use changes in maximal width and MLS rather than merely the postoperative measurements (Tables 7 and 8). Given the number of outcome events available, we also elected to examine unilateral cases only, resulting in 218 patient cohorts. We elected to present three models to demonstrate how the odds ratio changes. Model 1 demonstrates that lack of drain insertion and preoperative GCS are significantly associated with recurrence, as already reported in the main study [3]. Model 2 reveals a reduction in association of drain insertion with recurrence when taking change in maximal width into account. Model 3 demonstrates that the change in maximal MLS weakens the association between change in maximal width and recurrence. Similarly, in the analyses for unfavourable functional outcome, change in maximal width/shift appears to be a positive confounder for drain insertion. However, on multivariate analysis, these variables were not significantly associated with functional outcome.

**Table 7:** Adjusted odds ratios for recurrence within 60 days using multiple logistic regression model based on 218 patients

	Model 1		Model 2		Model 3	
	OR (95% CI)	*p* value	OR (95% CI)	*p* value	OR (95% CI)	*p* value
Drain inserted	0.36 (0.15–0.76)	0.03	0.68 (0.25–1.84)	0.45	0.71 (0.26–1.98)	0.51
Preoperative GCS (13–15)	0.34 (0.14–0.89)	< 0.01	0.35 (0.18–0.81)	0.01	0.34 (0.14–0.80)	0.01
Change in maximal width	-	-	0.90 (0.84–0.96)	< 0.01	0.95 (0.87–1.02)	0.17
Change in maximal shift	-	-	-	-	0.87 (0.77–0.99)	0.03

**Table 8:** Adjusted odds ratios for unfavourable functional outcome at discharge using multiple logistic regression model based on 218 patients

	Model 1		Model 2		Model 3	
	OR (95% CI)	*p* value	OR (95% CI)	*p* value	OR (95% CI)	*p* value
Age	1.06 (1.03–1.10)	< 0.01	1.07 (1.03–1.10)	< 0.01	1.06 (1.02–1.10)	< 0.01
Preoperative mRS	6.02 (2.63–13.8)	< 0.01	6.49 (2.80–15.1)	< 0.01	6.52 (2.80–15.2)	< 0.01
Preoperative GCS (13–15)	0.59 (0.28–1.28)	0.18	0.62 (0.28–1.33)	0.22	0.55 (0.25–1.22)	0.14
Drain inserted	0.24 (0.08–0.67)	< 0.01	0.32 (0.10–0.96)	0.04	0.33 (0.11–1.02)	0.05
> 1 burr hole	0.44 (0.14–1.46)	0.18	0.44 (0.14–1.45)	0.18	0.47 (0.14–1.53)	0.21
Postoperative bed rest						
1–12 h	1.32 (0.31–5.69)	0.71	1.42 (0.33–6.13)	0.64	1.56 (0.36–6.76)	0.56
12–24 h	1.94 (0.84–4.50)	0.12	1.84 (0.80–4.29)	0.16	1.90 (0.81–4.47)	0.14
24–48 h	2.97 (1.04–8.43)	0.04	2.69 (0.94–7.72)	0.07	2.63 (0.91–7.62)	0.08
> 48 h	4.48 (0.42–47.4)	0.21	4.16 (0.39–44.34)	0.24	3.99 (0.37–42.7)	0.25
Change in maximal width	-	-	0.96 (0.91–1.02)	0.16	0.99 (0.92–1.05)	0.66
Change in maximal shift	-	-	-	-	0.93 (0.84–1.02)	0.14

## Discussion

We have shown that the use of drains results in significantly improved postoperative imaging in burr hole evacuation for CSDH. We also found that change in maximal width and MLS were significant predictors of recurrence, and the former remained a significant predictor for functional outcome.

### Drains versus no drains

Few studies have examined the difference in postoperative radiology when evaluating drain efficacy. In one of the earliest prospective studies supporting the notion that drains reduce CSDH recurrence, change in haematoma volume was also evaluated. In their small study, Wakai et al. report those undergoing burr hole evacuation with drain insertion had a significantly more rapid reduction in haematoma volume at day 1 postoperatively, but differences were no longer evident after this [30]. In another small prospective study, the authors demonstrated higher resolution rate on the 5th postoperative day in burr hole evacuation with a drain group compared to burr hole evacuation alone (60% versus 40%); however, at 30 days, this difference no longer existed, and they also failed to demonstrate significant difference in recurrence between groups [8].

Our results clearly demonstrate that drains result in significantly greater change in subdural width and MLS in unilaterally operated haematomas and a non-significant greater change in subdural width in bilaterally operated haematomas. Early functional outcome also appears to be improved with the use of drains [3]. Given that change in subdural width and MLS were found to be associated with lower recurrence and improved functional outcome in unilateral operated patients it is likely that drains mediate their effect through more rapid improvement in size of subdural collection and mass effect on the brain, allowing for improved brain expansion. This is an intuitive and logical finding, and provides further evidence for the use of drains in surgical management of CSDH.

### Radiological predictors

We found postoperative width and MLS, alongside change in maximal width and MLS, to all be associated with recurrence rate and functional outcome. Our multivariate models suggest preoperative GCS remains the strongest prognostic factor for recurrence with very little change with the addition of radiological characteristics. This is open to interpretation, but one could postulate that placement of a drain increases the change in maximal width/shift thereby reducing the risk of recurrence.

A number of studies have tried to establish either directly or indirectly whether any particular pre- or postoperative radiological parameters give rise to a higher recurrence rate [6, 9, 14, 21, 26, 28, 29]; very few studies have sought to establish whether radiology correlates with functional outcome. Unfortunately, there is no consensus over which radiological findings predict higher recurrence and these results are marred by variable definitions of recurrence and timing of postoperative CT scanning, along with underpowered studies sometimes employing suboptimal choice of statistical tests. However, there is a growing body of evidence that suggests larger haematomas with more mass effect, both pre- and postoperatively, along with greater amounts of postoperative subdural air are all associated with increased recurrence rates.

Schwarz et al. found midline shift between 6 and 10 mm was a significant risk factor for reoperation [26]. Similarly, in a recent large Chinese study reporting on recurrence rates in burr hole evacuation for unilateral CSDH in 242 patients, the authors report that preop MLS > 10 mm was significantly associated with recurrence [21]. Stanisic et al. purport that preoperative haematoma volume and the residual total haematoma cavity volume on the 1st postoperative day after removal of the drainage were radiological predictors of recurrence [28]. In a larger study of 412 patients, it was found postoperative MLS along with preoperative subdural width was significantly associated with recurrence [6]. Tahsim-Oglou et al. when evaluating the role of prophylactic dose heparin in recurrence found that change in width of the haematoma from pre- and postop was significantly associated—the recurrence group had significantly lower median change in width [29].

### Functional outcomes

Ro et al. reported on predictors of early functional outcome at 3 months. In terms of radiological factors, they report that isodense haematomas were significantly associated with improved functional outcome; preoperative MLS did not influence functional outcome [22]. However, another group report that larger residual haematomas were significantly associated with worse functional outcome at 3 months. This factor did not quite reach significance for increased recurrence although the presence of ongoing mass effect on postoperative CT was significantly associated with recurrence [18]. Our data clearly demonstrate that change in maximal subdural width is associated with functional outcome. However, we are limited by lack of long-term follow-up and it would be of interest to see whether improved postoperative imaging is associated with superior functional outcome in the longer term. It has been shown that the use of subdural drains improves longer term survival [13].

### Strengths and limitations

This subgroup analysis comprises a large study on radiological predictors of recurrence and functional outcome in surgical treatment of CSDH, and in the evaluation of radiological changes in the presence and absence of drains. We achieved this through a highly co-ordinated data collection period across multiple hospitals in the UK. We collected a large amount of data on each patient and established which of these variables predict recurrence and functional outcomes; these were factored into the multivariate models to reduce effects on known confounders and provide robust statistical analysis.

Given the primary aim of this subgroup analysis was to assess differences in radiological outcomes between those patients who underwent drain insertion compared with those who did not have drains, we had to exclude a large number of patients who underwent preoperative CT scan only. We did, however, perform a supplementary analysis including these patients, and the results were very similar to the smaller group who underwent postoperative CT scan (see supplementary tables 3–8c). The main difference is that preoperative maximal width became significantly associated with recurrence on multivariate analysis (see supplementary table 8a), which is likely secondary to larger effect size with more patients leading to more power.

A further analysis demonstrated that there were minimal differences between the patients analysed in this study (those with both pre- and postoperative scans) and those patients excluded (those with preoperative imaging only; see supplementary table 2). As expected, all but recurrence were not significantly different between these two groups. It is, however, expected that the group with postoperative imaging would have a higher risk of recurrence given that imaging will have been requested when recurrence was suspected clinically.

Radiology was reviewed by neurosurgeons and/or radiologists in each centre without central independent review. Nonetheless, simple radiological parameters were chosen in an attempt to mitigate this issue. The analysis was post hoc and therefore should be viewed as exploratory. Although this was a prospective randomised study, there was no randomisation. The decision to perform postoperative imaging was also at the discretion of the surgical team, which could give rise to selection bias and prejudiced associations observed. The timing of postoperative CT scan was also variable although, importantly, not significantly different between the two groups; the median for both groups was 2 days postsurgery (*p* = 0.5). Also, the proportion of CT scans requested as ‘routine’ versus ‘for concern’ did not significantly differ between groups. We felt that it did not make sense to add scan interval and scan indication to our multivariate analyses as we are not trying to develop a predictive model for various outcomes, but rather to evaluate radiological markers.

The decision to proceed with revision surgery was at the discretion of the patient’s consultant neurosurgeon, based on clinical symptoms, correlated with imaging. The symptomatic recurrence rate we observed at 60 days may have underestimated the true rate if there were late recurrences, but previous studies have suggested that recurrence is most likely within this time frame [19]. The median time to recurrence within the study population agreed with that reported previously [2].

We found some significant baseline and perioperative differences between groups compared (‘drain’ versus ‘no drain’ group), namely group size, preoperative haematoma size and mRS score as well as age. The ‘no drain’ group was much smaller than those who received drains. This is a reflection of relatively current UK-wide practice; the imbalance was expected in view of level 1 evidence which supports the use of subdural drains [23]. Those that did not undergo drain insertion may well have had minimal room in the subdural space for safe drain placement. This is most common in younger patients with smaller haematomas and/or greater intraoperative brain re-expansion; these patients also generally tend to have higher preoperative functional status (Tables 1, 2 and 3), providing a plausible explanation to explain the baseline differences. It is difficult to know whether this unavoidable selection bias has influenced the findings of significant improved radiology in the drain group given their larger mean preoperative haematoma width (25 mm), although importantly those who did not receive drains still had sizeable clots (20 mm) preoperatively.

The aforementioned differences between ‘drain’ and ‘no drain’ groups will not have confounded our analysis of radiological predictors of recurrence and functional outcome, as this evaluated these groups as one. For all multivariate analyses performed, we selected variables that had been shown to be significantly associated with the respective outcomes, recurrence and early mRS score. In our index study, age, initial mRS, burr hole number, drain insertion and bed rest were significantly associated with functional outcome [3]. Single burr holes are generally reserved for older patients with greater comorbidities, where a shorter operation is perceived to be in the patient’s best interest. However, age, > 1 burr hole used and bed rest did not influence symptomatic recurrence rate; preoperative GCS and drain insertion were the only significant predictors [3].

### Implications for practice

Although of academic interest, the most important radiological variables associated with recurrence and functional outcome are those which we can manipulate clinically. We have shown that drains result in smaller postoperative subdural collections with less mass effect, and provide a plausible mechanism to reduce postoperative subdural air as they allow the operator to fill the subdural space wit0h saline prior to closure; there are some data that support reduced recurrence with less subdural air [20]. These data therefore provide further evidence that subdural drains should be employed, unless the brain has fully expanded preventing safe placement of a subdural drain. In such cases, an extra-calvarial subgaleal or subperiosteal drain can be left behind; a growing body of evidence suggests that these are also efficacious [5, 10, 12, 16, 27, 31]. The present data also suggest that the mechanism of action of subdural drains involves a reduction of the subdural collection width and the associated mass effect. Moreover, this provides a potential treatment target for adjuvant pharmacological therapies, such as atorvastatin [15] or tranexamic acid, in an attempt to further reduce recurrence, which in most modern series is around 10–15%.

Furthermore, one could argue a role for routine postoperative scanning of all patients undergoing drainage of CSDH, and those with smaller reductions in MLS and subdural size may require closer clinical follow-up over the subsequent months after index surgery. Should patients require further surgery a postoperative scan would also inform as to whether this is due to genuine recurrence or simply a large residual collection from index surgery. Nevertheless, we are also aware of recent evidence from a randomised trial which demonstrated no benefit for routine follow-up CT after surgery for CSDH compared to CT performed only in patients with clinical deterioration or persistent deficits [25].

## Conclusions

The use of drains in burr hole evacuation for CSDH is associated with improved postoperative imaging alongside lower recurrence rates and improved early functional outcomes. Overall, change in maximal width and MLS were significant predictors of recurrence, and the former remained a significant predictor for functional outcome.

### Collaborators

Afshari FT, Ahmed AI, Alli S, Al-Mahfoudh R, Bal J, Belli A, Borg A, Bulters D, Carleton-Bland N, Chari A, Coope D, Coulter IC, Cowie CJ, Critchley G, Dambatta S, D’Aquino D, Dhamija B, Dobson G, Fam MD, Gray WP, Gregson BA, Grover PJ, Halliday J, Hamdan A, Hill CS, Jamjoom AAB, Joannides AJ, Jones TL, Joshi SM, Kailaya-Vasan A, Karavasili V, Khan SA, King AT, Kuenzel A, Livermore LJ, Lo W, Martin J, Matloob S, Mitchell P, Mowle D, Narayanamurthy H, Nelson RJ, Ngoga D, Noorani I, O’Reilly G, Othman H, Owusu-Agyemang K, Manjunath, Marcus H, Prasad KS, Plaha P, Pollock J, Prasad KS, Price R, Pringle C, Ray A, Reaper J, Scotton W, Shapey J, Simms N, Smith S, Statham P, Steele L, St George J, Stovell MG, Tarnaris A, Teo M, Thomson S, Thorne L, Vintu M, Whitfield P, Wilson M, Wilby M, Woodfield J, Zaben M. We kindly request the above collaborators to be cited on PubMed as collaborators.

## Electronic supplementary material

ESM 1 (PDF 207 kb)
