# Supplementary material for: Drains result in greater reduction of subdural width and midline shift in burr hole evacuation of chronic subdural haematoma
Source: Acta Neurochir (Wien). 2020 Apr 27;162(6):1455–66. doi: 10.1007/s00701-020-04356-z (PMC7235065; doi:10.1007/s00701-020-04356-z)
Supplement: Supplementary file 1 — (PDF 207 kb) [file 701_2020_4356_MOESM1_ESM.pdf]

Supplementary table 1 Number of patients contributed per NSU

Hospital	Patient number (n=319)	Percentage	Cumulative Percentage
1	19	5.96	5.96
2	18	5.64	11.6
3	17	5.33	16.93
4	34	10.66	27.59
5	26	8.15	35.74
6	24	7.52	43.26
7	8	2.51	45.77
8	5	1.57	47.34
9	18	5.64	52.98
10	19	5.96	58.93
11	31	9.72	68.65
12	8	2.51	71.16
13	2	0.63	71.79
14	2	0.63	72.41
15	14	4.39	76.8
16	1	0.31	77.12
17	5	1.57	78.68
18	4	1.25	79.94
19	13	4.08	84.01
20	5	1.57	85.58
21	7	2.19	87.77
22	13	4.08	91.85
23	7	2.19	94.04
24	2	0.63	94.67
25	8	2.51	97.18
26	9	2.82	100

Supplementary table 2 Comparison of pertinent characteristics of 683 patients who underwent burr-hole drainage of CSDH with and without postoperative imaging

	No postop imaging	Postop imaging	P value
Number of patients	364	319	
Age (median; IQR)	78 (69-85)	77 (66-84)	0.46
Pre-op GCS			0.5
3-8	9 (2.5%)	17 (5.3%)	
9-12	46 (12.6%)	41 (12.9%)	
13-15	309 (84.9%)	309 (84.9%	
Time to operation, median (IQR)	1 (1-3)	1 (1-3)	0.73
Drain Insertion			0.47
No	59 (16.2%)	45 (14.2%)	
Yes	305 (83.8%)	272 (85.8%)	
Pre-operative maximum width in mm, mean (SD)	25.4 (10.5)	26.1 (11.1)	0.4
Pre-operative maximum MLS in mm, mean (SD)	8.2 (6.5)	7.9 (4.9)	0.56
Recurrence	23 (6.5%)	38 (12.2%)	0.012

Supplementary table 3 Baseline characteristics of 683 patients who underwent burr-hole drainage of CSDH (including patients with pre-operative scans only)

	Total (n=319)
Age (median; IQR)	77 (67-84)
Gender	
Female	219 (32.1)
Male	464 (67.9)
Medical comorbidities	
Diabetes mellitus	113 (16.5)
Dementia	76 (11.1)
COPD	37 (5.4)
Cerebrovascular event	110 (16.1)
Ischaemic heart disease	173 (25.3)
Arrhythmia	138 (20.2)
Epilepsy	30 (4.4)
CSF shunt	7 (1.0)
Malignancy	59 (8.6)
Metallic heart valve	14 (2.1)
History of head injury in past 3 months	427 (62.5)
Prior antithrombotic use	285 (41.7)
Prior antiplatelet use	161 (23.6)
Prior warfarin use	125 (18.3)
Other antithrombotics	11 (1.6)

Supplementary table 4 Perioperative characteristics of 683 patients who underwent burr-hole drainage of CSDH (including patients with pre-operative scans only)

	Total (n=319)
Pre-operative platelet transfusion	64 (9.4)
Pre-operative vitamin K	108 (15.8)
Pre-operative FFP	14 (2.1)
Pre-operative GCS (median, IQR)	14 (14-15)
GCS 3-8	26 (3.8)
GCS 9-12	87 (12.7)
GCS 13-15	570 (83.5)
Pre-operative mRS	
mRS 0-3	400 (58.6)
mRS 4-5	283 (41.4)
CSDH laterality	
Left	270 (39.5)
Right	238 (34.9)
Bilateral	158 (23.1)
Unknown/missing	17 (2.5)
Timing of operation	
Within 2 days of referral	481 (70.4)
After 2 days of referral	181 (26.5)
Unknown/missing	21 (3.1)
Number of burr hole(s)	
Single burr hole	55 (8.1)
>1 burr holes	626 (91.7)
Unknown/missing	2 (0.3)
Post-operative bed rest	
No specific instructions	271 (39.7)
1-12 hours	46 (6.7)
12-24 hours	256 (37.5)
24-48 hours	99 (14.5)
48+ hours	9 (1.3)
Unknown/missing	2 (0.3)
Post-operative imaging*	
Routine	184 (58.0)
Due to concerns	133 (42.0)

*318 patients had post-operative imaging

Supplementary table 5 Radiological characteristics of 683 patients who underwent burr-hole drainage of CSDH (including patients with pre-operative scans only)

	Total* (n=683)	Unilateral (n=508)	Bilateral (n=158)
CSDH density on initial CT scan			
Hypodense	209 (30.1)	153 (30.1)	50 (31.7)
Isodense	138 (20.2)	103 (20.3)	31 (19.6)
Mixed	336 (49.2)	252 (49.6)	77 (48.7)
Presence of membrane on CT scan			
Yes	259 (37.9)	197 (38.8)	56 (35.4)
No	424 (62.1)	311 (61.2)	102 (64.6)
Pre-operative maximal width (mm)	25 (18-30)	22 (17-28)	35 (25-44)
Pre-operative midline shift (mm)†	8 (5-11)	9 (6-12)	3 (0-6)
Time to operation (days)	1 (1-3)	1 (1-2.5)	2 (1-3)
Days between operation and post-operative scan (median; IQR)	2 (1-4)	2 (1-4)	2 (1-4)
Post-operative maximal width (mm)	14 (9-21)	13 (8-19)	20 (12-29)
Change in maximal width (mm)			
Median (IQR)	10 (5-15)	9 (5-14)	13 (7-20)
Reduced width	288 (90.9)	202 (90.6)	79 (91.9)
Same or increased width	29 (9.2)	21 (9.4)	7 (8.1)
Change in midline shift (mm)			
Median (IQR)	4 (1-6)	5 (2-7)	2 (0-4)
Reduced shift	256 (80.8)	192 (86.1)	56 (65.1)
Same or increased shift	61 (19.2)	31 (13.9)	30 (34.9)

*17 patients had missing data on operative laterality
†Maximal width in bilateral cases is the sum of the maximal width on each side

Supplementary table 6 Recurrence and Functional Outcome in 683 patients who underwent burr-hole drainage of CSDH (including patients with pre-operative scans only)

	Total* (n=683)	Unilateral (n=508)	Bilateral (n=158)
Recurrence			
No recurrence	604 (88.4)	451 (88.8)	139 (88.0)
Recurrence occurred	61 (8.9)	44 (8.7)	15 (9.5)
Unknown/missing	18 (2.6)	13 (2.6)	4 (2.5)
Discharge functional status (mRS)			
mRS 0-3	516 (75.6)	381 (75.0)	119 (75.3)
mRS 4-6	149 (21.8)	114 (22.4)	35 (22.2)
Unknown/missing	18 (2.6)	13 (2.6)	4 (2.5)

*17 patients had missing data on operative laterality

Supplementary table 7 Unadjusted odds ratio for recurrence and unfavourable functional outcome at discharge in 683 patients who underwent burr-hole drainage of CSDH (including patients with pre-operative scans only)

	Recurrence			Unfavourable functional outcome		
	OR	95% CI	P-value	OR	95% CI	P-value
Density on CT						
Hypodense	Ref	-	-	Ref	-	-
Isodense	0.95	0.43-2.11	0.91	0.45	0.25-0.81	0.01
Mixed	1.20	0.65-2.21	0.57	0.81	0.54-1.22	0.32
Presence of membrane on CT scan	1.32	0.77-2.24	0.31	0.96	0.66-1.40	0.83
Pre-operative maximal width	1.02	1.00-1.05	0.06	1.01	0.99-1.02	0.51
Pre-operative maximal shift	1.01	0.97-1.06	0.63	1.00	0.97-1.03	0.80
Post-operative maximal width	1.07	1.04-1.11	<0.01	1.03	1.00-1.05	0.04
Post-operative maximal shift	1.28	1.18-1.40	<0.01	1.13	1.06-1.20	<0.01
Change in maximal width	0.92	0.88-0.95	<0.01	0.95	0.92-0.98	<0.01
Change in maximal shift	0.86	0.79-0.93	<0.01	0.93	0.88-0.99	0.02

Supplementary table 8a Adjusted odds ratios for recurrence within 60 days using multiple logistic regression model based on 644 patients who underwent burr-hole drainage of CSDH with complete data (including patients with pre-operative scans only)

	OR	95% CI	P-value
Drain inserted	0.39	0.21-0.73	<0.01
Pre-operative GCS (13-15)	0.43	0.24-0.77	<0.01
Pre-operative maximal width (mm)	1.03	1.00-1.05	0.02
Pre-operative maximal shift (mm)	1.01	0.97-1.05	0.72

Supplementary table 8b Adjusted odds ratios for functional outcome at discharge using multiple logistic regression model based on 644 patients who underwent burr-hole drainage of CSDH with complete data (including patients with pre-operative scans only)

	OR	95% CI	P-value
Age	1.08	1.05-1.10	<0.01
Pre-operative mRS (mRS 4-5)	4.05	0.53-6.47	<0.01
Pre-operative GCS (13-15)	0.38	0.24-0.62	<0.01
Drain inserted	0.53	0.29-0.98	0.04
>1 burr-hole	0.45	0.22-0.94	0.03
Post-operative bed rest			
1-12 hours	1.19	0.46-3.09	0.72
12-24 hours	1.39	0.85-2.29	0.19
24-48hrs	1.54	0.82-2.90	0.18
>48hrs	7.41	0.93-59.3	0.06
Pre-operative maximal width (mm)	0.99	0.97-1.01	0.37
Pre-operative maximal shift (mm)	0.98	0.94-1.03	0.45

Supplementary table 8c Adjusted odds ratios for functional outcome at discharge using multiple logistic regression model based on 644 patients who underwent burr-hole drainage of CSDH with complete data (including patients with pre-operative scans only); this includes clot density as a variable

	OR	95% CI	P-value
Age	1.08	1.05-1.10	<0.01
Pre-operative mRS (mRS 4-5)	3.99	2.49-6.39	<0.01
Pre-operative GCS (13-15)	0.38	0.23-0.62	<0.01
Density on initial CT scan			
Hypodense	Ref	-	-
Isodense	0.66	0.34-1.28	0.34
Mixed	0.67	0.41-1.08	0.41
Drain inserted	0.56	0.30-1.04	0.07
>1 burr-hole	0.46	0.22-0.96	0.04
Post-operative bed rest			
1-12 hours	1.13	0.43-2.94	0.81
12-24 hours	1.35	0.82-2.23	0.24
24-48hrs	1.60	0.84-3.02	0.15
>48hrs	7.68	0.98-60.2	0.05
Pre-operative maximal width (mm)	0.99	0.97-1.01	0.48
Pre-operative maximal shift (mm)	0.98	0.94-1.03	0.52
